# Isolation of a structural intermediate during switching of degree of interpenetration in a metal–organic framework†
†Electronic supplementary information (ESI) available: Synthetic procedure, detail physical methods, detailed crystallographic information, additional figures, Rietveld refinements, CO_2_ sorption isotherm, additional XRPD patterns. CCDC 1052168–1052173. For ESI and crystallographic data in CIF or other electronic format see DOI: 10.1039/c5sc01796c


**DOI:** 10.1039/c5sc01796c

**Published:** 2015-06-11

**Authors:** Himanshu Aggarwal, Raj Kumar Das, Prashant M. Bhatt, Leonard J. Barbour

**Affiliations:** a Department of Chemistry and Polymer Science , University of Stellenbosch , Matieland 7602 , Stellenbosch , South Africa . Email: ljb@sun.ac.za

## Abstract

A known pillared layered metal–organic framework [Co_2_(ndc)_2_(bpy)] is shown to undergo a change in degree of interpenetration from a highly porous doubly-interpenetrated framework (**2fa**) to a less porous triply-interpenetrated framework (**3fa**). The transformation involves an intermediate empty doubly-interpenetrated phase (**2fa′**) which has been isolated for the first time for this kind of phenomenon by altering the conditions of activation of the as-synthesized material. Interestingly, all the transformations occur in single-crystal to single-crystal fashion. Changes in degree of interpenetration have not been explored much to date and their implications with regard to the porosity of MOFs still remain largely unknown. The present study not only provides a better understanding of such dramatic structural changes in MOF materials, but also describes an original way of controlling interpenetration by carefully optimizing the temperature of activation. In addition to studying the structural mechanism of conversion from **2fa** to **3fa**, sorption analysis has been carried out on both the intermediate (**2fa′**) and the triply-interpenetrated (**3fa**) forms to further explain the effect that switching of interpenetration mode has on the porosity of the MOF material.

## Abstract 2

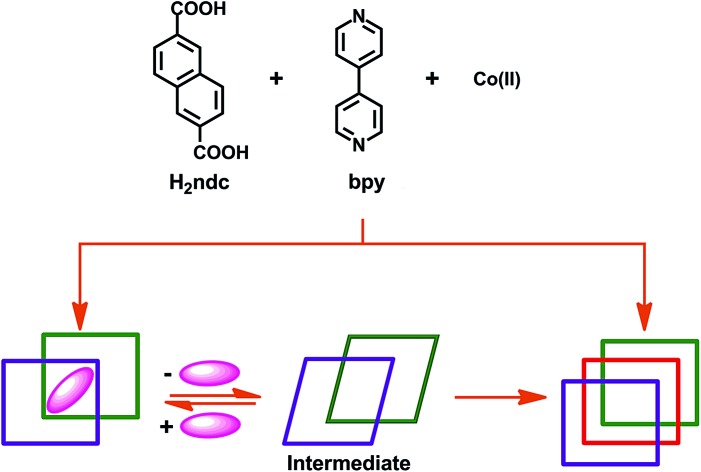
A structural intermediate has been isolated for the first time during switching of interpenetration from twofold to threefold in the MOF [Co_2_(ndc)_2_(bpy)].

## Introduction

Owing to their high surface areas and porosity, metal–organic frameworks (MOFs) have been recognized as promising materials for gas sorption^1^ and the separation^2^ of substances.^3^ Many groups have tried to enhance the surface areas of MOFs by increasing the length of the organic bridging ligands.^4^ However, the presence of larger spaces in a framework frequently leads to network catenation, also known as interpenetration.^5^ On the one hand, interpenetration promotes framework flexibility and guest selectivity^6^ in MOFs, but on the other hand it also results in lower surface areas and void volumes.^7^ Several reports have demonstrated that the degree of interpenetration can be controlled for certain MOFs by varying the conditions for crystallization such as choice of solvent,^8^ temperature,^9^,^10^ concentration,^10^*etc.*

Over the past few years many new MOFs have been prepared by successfully employing methodologies^8^–^10^ for controlling the degree of interpenetration in order to obtain less dense (or more porous) frameworks. In a typical study, a MOF is first synthesized (mostly using solvothermal methods) and then activated/desolvated by heating at a relatively high temperature in order to obtain the guest-free form of the as-synthesized material. The activated sample is usually expected to retain its host framework integrity upon loss of solvent molecules from the channels. In some cases the activated material retains sufficient macroscopic integrity such that its structure can be elucidated by means of single crystal X-ray diffraction. However, in most instances the single crystals do not survive activation intact and the structure of the activated material therefore remains unknown.^11^ Typically, in such cases thermogravimetric analysis (TGA) is used to confirm complete removal of guest molecules from the channels and the structural integrity of the host framework is inferred from X-ray powder diffraction (XRPD). Sorption data are then recorded with the assumption that the activated form has the identical (or at least similar) structure to that of the host framework of the as-synthesized material. Most of the time the sorption results are reasonable but several reports have mentioned unexpected loss of porosity upon activation of MOFs.^12^–^16^

Since our first report on the change in degree of interpenetration of a known MOF,^17^ we have been interested in investigating this surprising phenomenon in more detail with a view to elucidating underlying mechanisms and to rationalizing the effect of such an extreme change on the porosity of other MOF materials. Since interpenetrated frameworks involve two or more independent grids with no formal bonds between them, they cannot be disentangled without breaking metal–ligand coordination bonds. “Soft” secondary building units (SBUs) have been reported^18^ that involve partial breaking and formation of metal-chelate coordination bonds. However, the complete detachment of metal–ligand linkages requires a relatively large amount of energy, and thus any change in interpenetration mode is generally assumed to be quite unlikely. Indeed, there have been only a few reports of changes in interpenetration, either as a result of removal of coordinated solvent^19^ upon heating at elevated temperatures, or by loss of solvent of crystallization^17^,^20^ upon activation of crystals. Furthermore, the transformations generally involve concomitant degradation of crystal singularity. In order to better understand the mechanisms that govern solid-state phase transformations at the molecular level, and to rule out any possibility of recrystallization *via* an amorphous phase, monitoring such processes as single-crystal to single-crystal transformations is very important.^21^

Recently, we showed that two doubly pillared layered Cd(ii) non-interpenetrated MOFs, [Cd(tp)(4,4′-bpy)] and [Cd(atp)(4,4′-bpy)], (tp = terephthalate; 4,4′-bpy = 4,4′-bipyridine and atp = 2-aminoterephthalate) convert to doubly-interpenetrated frameworks upon loss of solvent molecules from the channels.^20^ Prior to that, we also showed that a doubly-interpenetrated MOF [Zn_2_(ndc)_2_(bpy)] (ndc = 2,6-napthalene dicarboxylate, bpy = 4,4′-bipyridyl) changes to its triply-interpenetrated form upon loss of solvent under ambient conditions, and that the transformation occurs in single-crystal to single-crystal fashion.^17^ The suggested mechanism involves bending of metal–ligand linkages with concomitant sliding of layers, ultimately resulting in concerted bond breaking and remaking to yield a new structure. It is possible that the transformation proceeds *via* an ‘empty’ doubly-interpenetrated phase that is crystallographically distinct from the as-synthesized material but, owing to the rapid transformation rate we were unable to observe any evidence for such an intermediate. Therefore it was not known whether the loss of solvent is concomitant with the change of interpenetration mode or if the transformation occurs in a stepwise fashion. In order to monitor the phenomenon in greater detail and to obtain more convincing evidence for the proposed mechanism, we have selected the analogous but more robust framework [Co_2_(ndc)_2_(bpy)] for further study. Owing to the crystal field stabilization energy of Co(ii), we expected the bond breaking and remaking process to be much slower in this particular case, thus enabling us to isolate an intermediate empty doubly-interpenetrated structure.

## Results and discussion

**Fig. 1:**
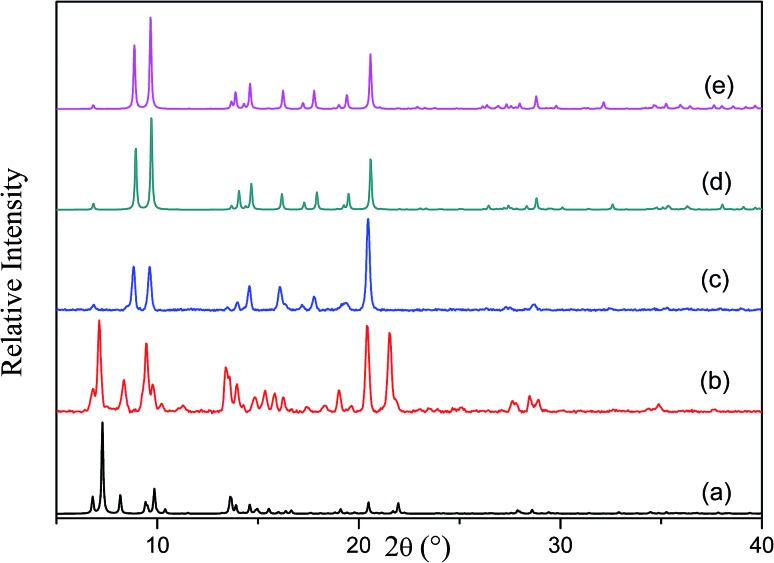
XRPD patterns of (a) **2fa** simulated from the crystal structure, (b) as-synthesized **2fa** crystals, (c) **2fa** crystals after heating at 120 °C under dynamic vacuum, (d) **3fa** simulated from the crystal structure and (e) **3f** [Zn_2_(ndc)_2_(bpy)] simulated from the crystal structure.

The [Co_2_(ndc)_2_(bpy)] framework has already been reported to form both doubly-12 and triply-interpenetrated^22^ structures under different conditions. Both of these forms have a three dimensional pillared layer structure containing Co_2_(COO)_2_ paddle-wheel secondary building units with 6-connected *pcu* net topology. Doubly-interpenetrated networks are quite well-known for this topology, whereas analogous triply-interpenetrated frameworks are far less common.5c,6b,6c,^22^ The doubly-interpenetrated framework has high ‘virtual porosity’,^23^ with *N*,*N*′-dimethylformamide (DMF) and water molecules occupying the channels, whereas the solvent-free triply-interpenetrated form is considerably less porous. To investigate a possible change in interpenetration upon activation, we first synthesised the twofold-interpenetrated (**2fa**) framework using the reported procedure.^12^ The phase purity of the as-synthesized crystals was confirmed by comparing the X-ray powder diffraction (XRPD) patterns of the bulk sample with the simulated pattern of the reported doubly interpenetrated form (Fig. S1†).^12^ The crystal structure of **2fa** contains 40% guest-accessible volume (calculated by Platon),^24^ with one water and three DMF molecules per asymmetric unit, although the solvent molecules could not be modelled. Thermogravimetric analysis (TGA) shows a steady weight loss between room temperature and 120 °C, followed by a single step decomposition beyond 400 °C (Fig. S2†). Based on the TGA results, crystals were activated at 120 °C under dynamic vacuum for 12 hours. The XRPD diffractogram of the activated form was significantly different from that of the as-synthesized crystals but similar to those simulated for triply-interpenetrated [Co_2_(ndc)_2_(bpy)]^22^ and the known threefold-interpenetrated structure of [Zn_2_(ndc)_2_(bpy)] (**3f**) (Fig. 1).^17^

Although most of the crystals became opaque upon desolvation, several single crystals were observed to remain intact. Single-crystal diffraction (SCD) analysis of these crystals yielded the threefold-interpenetrated structure already reported in the literature.^22^ This observation was consistent with our previous finding that a doubly-interpenetrated structure can convert to its triply-interpenetrated form upon removal of the guest molecules, and suggested that the occurrence of such a change in single-crystal to single-crystal fashion is not limited to only one system. In order to unequivocally rule out the possibility that a small number of threefold-interpenetrated crystals had not already been present before activation, we undertook to monitor the transformation using only one crystal, and to preserve its diffraction quality by activating under the mildest possible conditions.

**Fig. 2:**
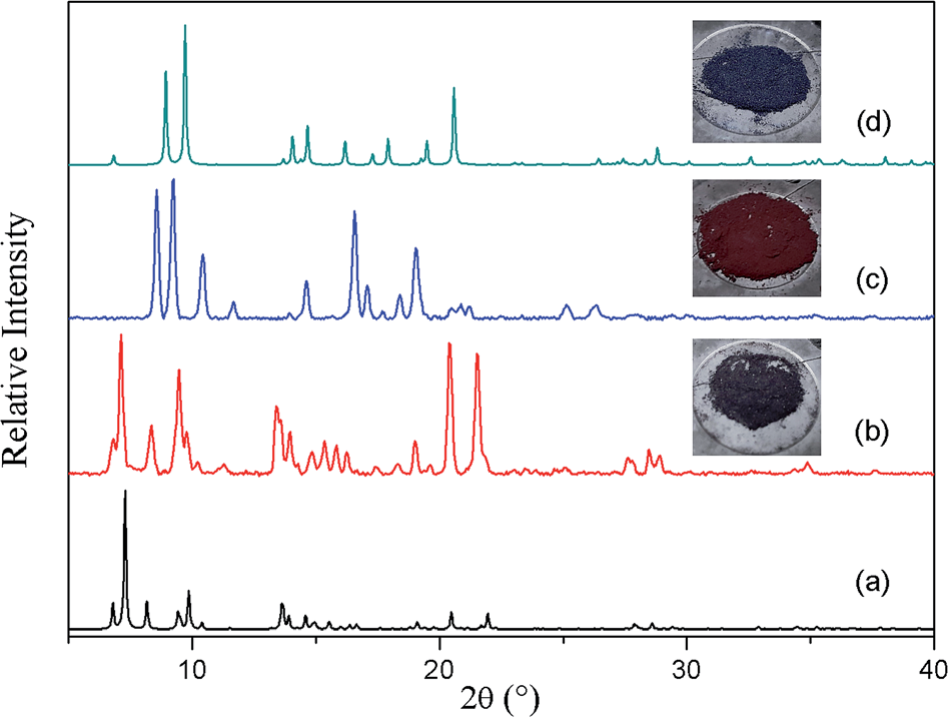
XRPD patterns of (a) **2fa** simulated from the crystal structure, (b) as-synthesized crystals of **2fa**, (c) **2fa** crystals after heating at 80 °C under dynamic vacuum, (d) **3fa** simulated from the crystal structure.

To slow down the activation process, a single crystal was allowed to lose its solvent of crystallization simply by exposure to atmospheric conditions (its unit cell parameters were monitored at regular intervals to follow the progress of the transformation). After almost one week X-ray diffraction analysis revealed that the crystal had converted to its triply-interpenetrated form (**3fa**). Note that, in the case of [Zn_2_(ndc)_2_(bpy)], conversion of **2f** to **3f** took place in only 6 hours at room temperature.^17^ We further established that complete conversion of bulk **2fa** to **3fa** at room temperature required about 10–12 days (Fig. S3†), thus verifying that conversion of the cobalt complex is much slower than that of its zinc analogue, as we had anticipated. We therefore explored the possibility of isolating an intermediate structure with a view to obtaining more insight into the mechanism of transformation from **2fa** to **3fa**. When as-synthesized crystals were activated at 80 °C under dynamic vacuum for 12 hours, the XRPD pattern of the resultant material was substantially different from those simulated from the crystal structures of the doubly- and triply-interpenetrated forms (Fig. 2).

**Fig. 3:**
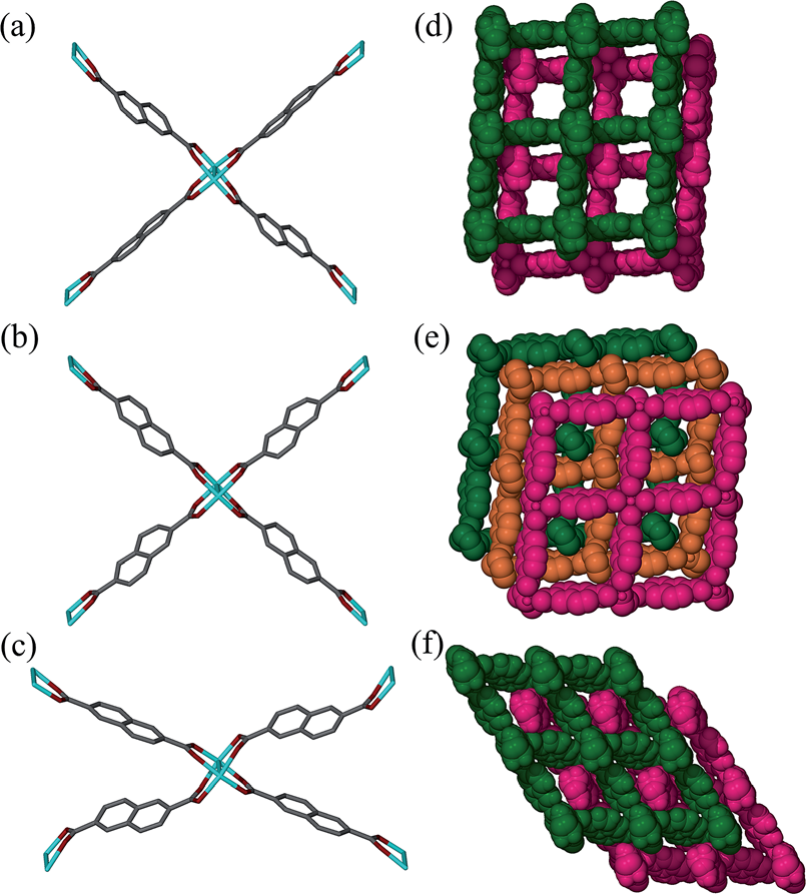
Perspective view showing Co_2_(COO)_4_ paddle-wheel linkages in (a) **2fa**, (b) **3fa** and (c) **2fa′** and packing diagrams of (d) **2fa** (guest molecules omitted), (e) **3fa** and (f) **2fa′**.

Surprisingly, SCD analysis of activated crystals taken from the bulk sample revealed a new doubly-interpenetrated structure (**2fa′**) devoid of DMF and water molecules (Fig. S4†), as also confirmed by TGA (Fig. S5†). The XRPD pattern of the activated crystals was compared with that simulated from the single-crystal structure to confirm the phase purity of the bulk sample (Fig. S6†). The structure of **2fa′** retains the connectivity of **2fa**, but, in order to compensate for the empty spaces resulting from guest removal, **2fa′** experiences extreme distortion of the metal–ligand linkages (Fig. 3c). As a result, the solvent accessible volume is reduced to 18%.^24^

**Fig. 4:**
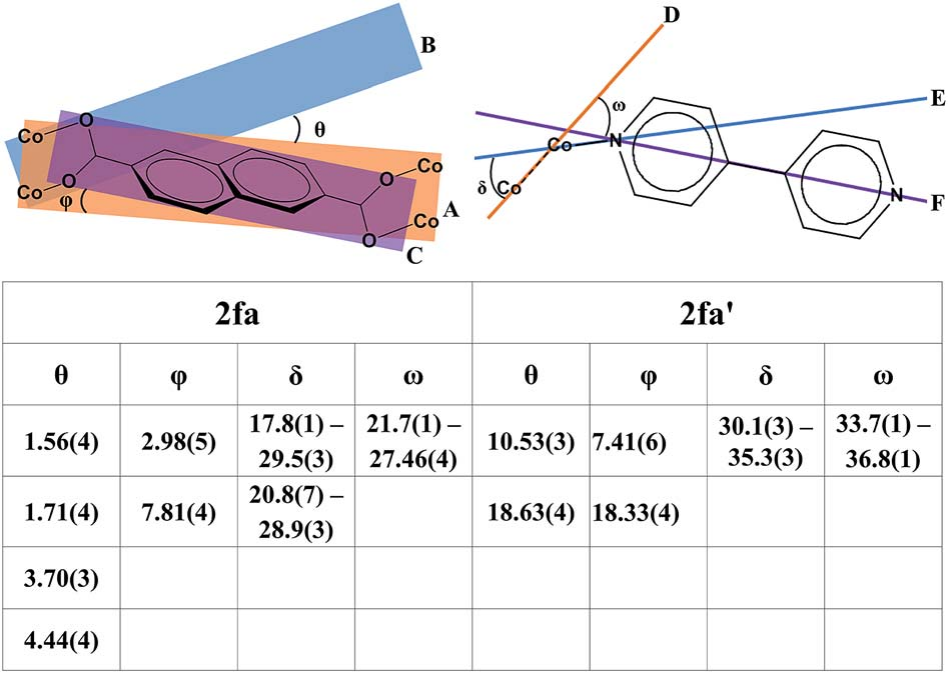
Top: diagrammatic representation of key dihedral and torsion angles in **2fa** and **2fa′**. A: Co_2_···Co_2_ metal atom plane; B: Co_2_–carboxylate oxygen plane; C: aromatic plane of ndc; D: Co_2_ cluster metal–metal axis; E: metal–nitrogen; F: N···N axis of bpy. Both crystallographic symmetry and geometrical disorders have been considered in measuring these parameters. Bottom: table of relevant angles in °.

Although the metal–ligand linkages involving both ndc and bpy are clearly bent, distortion of the former is more pronounced. This can be corroborated by examining the dihedral angles between the metal (*i.e.* Co_2_···Co_2_) plane and the metal-carboxylate plane (*θ*), as well as the metal plane and the aromatic plane of ndc (*φ*) in both **2fa** and **2fa′** (Fig. 4). The dihedral angles increase sharply upon conversion from **2fa** to **2fa′**.

In the case of bpy, a similar assessment was made by measuring the torsion angle (*δ*) between the Co_2_ cluster metal–metal axis and the Co–N linkage, and the torsion angle (*ω*) between the metal–metal axis and the aromatic part of bpy (Fig. 4). These angles are larger for **2fa′**, thus indicating bending of the metal–ligand linkages upon activation.

It is also clear from the measurements that the distortion of the (Co···Co)–ndc–(Co···Co) linkage is much greater than that of Co–bpy–Co, suggesting that the ndc linkers are in a more strained configuration. With reference to Fig. 3c, The structure of **2fa′** is comparable to that of the well-known MOF [Zn_2_(bdc)_2_(bpy)] which, upon activation, yields a considerably distorted and less porous phase (but owing to steric factors, further conversion to a more highly interpenetrated form is improbable in that case).^25^ Since ndc is longer than bdc, we observe even more distortion in the case of **2fa′**, as might be expected.

In our previous report describing a change in the degree of interpenetration for the [Zn_2_(ndc)_2_(bpy)] system we suggested a plausible mechanism for the transformation based on the available information:^17^ the metal–ligand bonds should bend on solvent removal in order to minimize the free space in the structure and this distortion of the framework should have two consequences: (1) the metal–ligand bonds will become weaker and easier to break and (2) adjacent layers from two independent networks will move closer to each other. When the metal–ligand bonds become sufficiently bent and weakened, they will break and form more linear and stronger bonds with suitably located metal centers of another network. This triggers a cascade of further bond breaking and formation, ultimately resulting in transformation of **2fa** to **3fa**. For the following reasons we suggested bpy as the more plausible choice than ndc to become involved in bond breaking and making: (1) at each end bpy coordinates in a monodentate fashion while bidentate chelation by ndc is associated with greater binding energy and (2) in the analogous [Zn_2_(bdc)_2_(bpy)] system the metal–bpy–metal linkage has indeed been observed^25^ to undergo severe distortion in order to minimize free space in the structure.

**Fig. 5:**
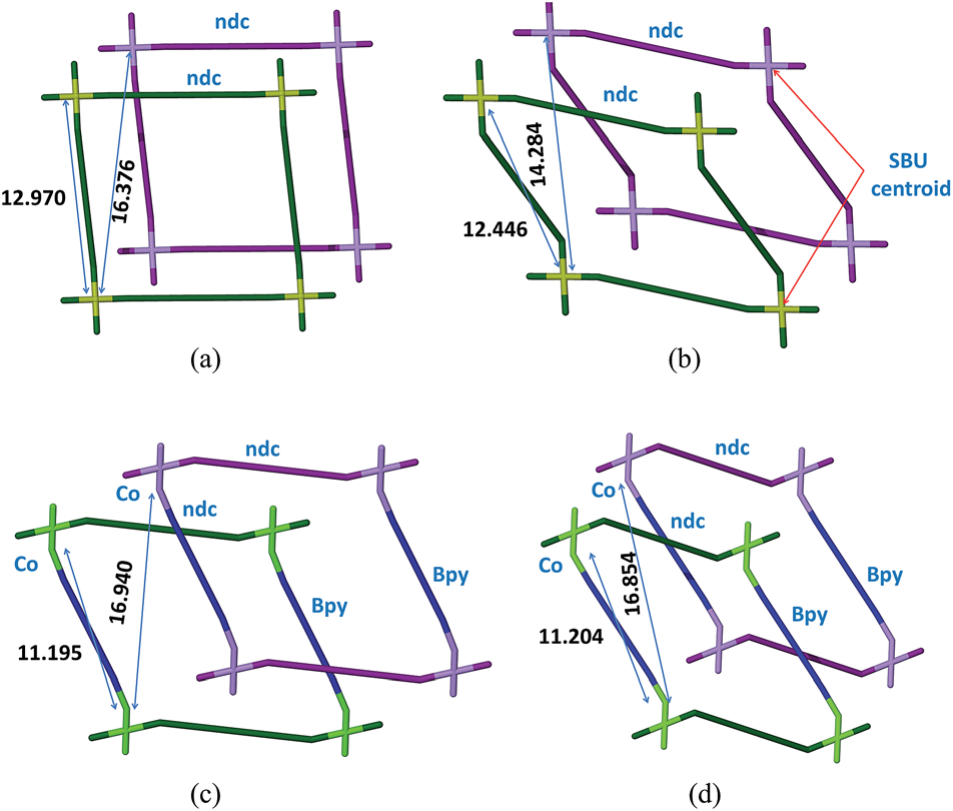
Schematic representation showing extent of bending and internodal distances for ndc and bpy; (a and b) **2fa** and **2fa′** viewed perpendicular to bpy, respectively and (c and d) **2fa** and **2fa′** viewed perpendicular to ndc, respectively.

Notwithstanding the above, isolation of the intermediate structure **2fa′** provides more insight into the possible mechanism of transformation. It is clear from the structure of **2fa′** that the ndc linkage undergoes more severe distortion than does bpy. Furthermore, ndc is geometrically better placed than bpy to form a new attachment to a neighboring metal cluster of another network. There are two symmetry-independent ndc ligands in the structure of **2fa**; one of them forms a comparatively straight connection between two Co_2_ clusters, with a node···node distance between two paddle-wheel units of 13.130 Å, while the other forms a relatively bent connection with a corresponding distance of 12.970 Å.‡
‡In the case of ndc, the node···node distances between two paddle-wheel units is taken as the distance between the Co–Co centroids of the two metal clusters bridged by the ndc ligand. The same protocol was used to determine the shortest distance between two paddle-wheel nodes of different networks. In the case of bpy, the distance is measured between the two Co metal centers to which the ligand is attached. It should be noted that only the geometrically favorable Co centers are taken in to account while measuring the shortest distances between paddle-wheel units of different networks. The shortest distance between two favorably oriented paddle-wheel nodes of different networks is 16.376 Å. Upon conversion to **2fa′** the corresponding Co···Co distances decrease to 13.025 Å and 12.446 Å, respectively. However, the latter ndc molecule could conceivably form a relatively straight connection between two paddle-wheel nodes of different networks without requiring any other major structural change; the distance between geometrically favorable paddle-wheels is only 14.284 Å (Fig. 5). On the other hand, in the case of bpy the distance between connected paddle-wheels is 11.195 Å in **2fa** and 11.204 Å in **2fa′**, whereas the shortest alternative distance (*i.e.* between paddle-wheel units of different frameworks) is 16.940 Å in **2fa** and 16.854 Å in **2fa′**.

The Co–O distances become elongated whereas the Co–N distances remain almost constant in **2fa′**, indicating weakening of metal carboxylate bonds as a direct consequence of activation, suggesting that **2fa′** is less stable than **2fa** (Table S1†). Upon further heating, it is reasonable to expect the Co–O bonds to become even weaker, ultimately leading to cleavage of the metal–ligand linkages involving ndc, followed by reattachment to energetically more favorable metal centers to yield an almost linear (Co···Co)–ndc–(Co···Co) linkage, and thus a triply-interpenetrated network. Although we do not rule out the possibility of simultaneous breaking of bpy linkers during this process, we believe it to be far less likely.

Having isolated the guest-free doubly-interpenetrated structure **2fa′**, we investigated whether it will convert to **3fa** (*i.e.* with the implication that **2fa′** is an intermediate phase during conversion from **2fa** to **3fa**). The same crystal was exposed to ambient conditions for several days after which single-crystal diffraction data were recollected. We thereby established that the crystal of **2fa′** had indeed converted to **3fa** in single-crystal to single-crystal fashion, thus demonstrating that **2fa′** is a metastable phase that ultimately converts to more stable **3fa**. Interestingly, the structure of **3fa** contains 16% solvent-accessible volume^24^ with no guest molecules in the channels. When bulk **2fa′** crystals were immersed in fresh DMF for 24 hours, the resulting XRPD pattern was similar to that of **2fa**, showing that the transformation from **2fa** to **2fa′** is reversible (Fig. S7†). When **3fa** crystals were immersed in fresh DMF we observed no change in the XRPD patterns. We also heated **3fa** crystals in fresh DMF at 120 °C for 48 hours, but the resulting XRPD pattern remained unchanged. This suggests that the transformation from **2fa** to **3fa** is irreversible under these conditions (Fig. S8†).

**Scheme 1:**
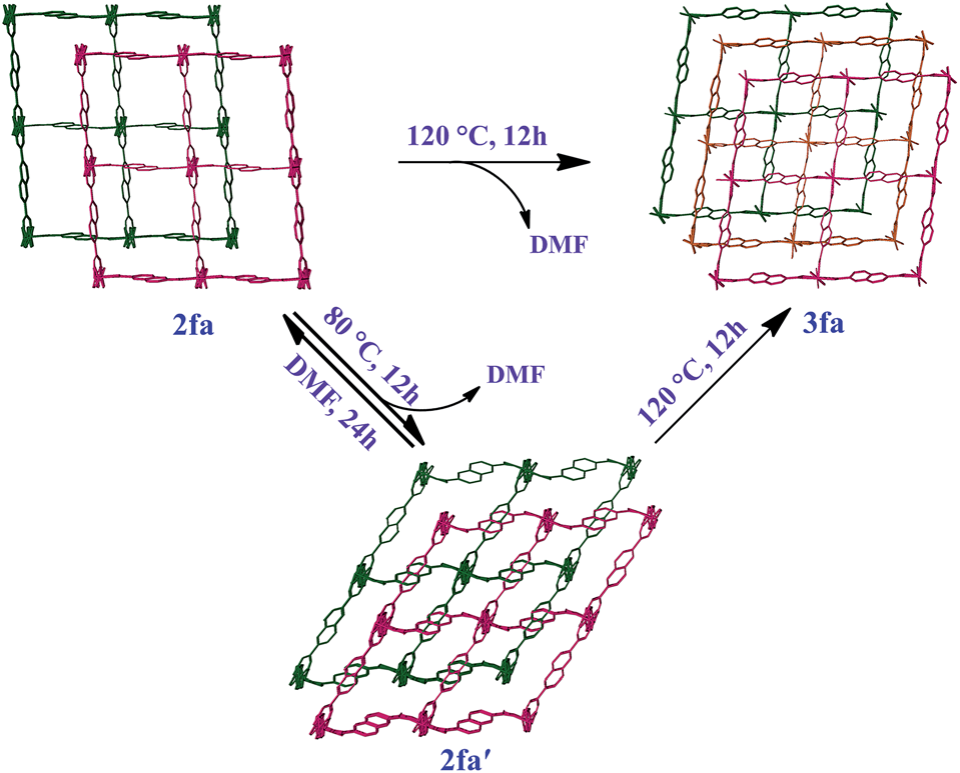
Schematic representation showing bending and switching of interpenetration in the [Co_2_(ndc)_2_(bpy)] system.

To verify unequivocally that **2fa** converts to **3fa***via* the structural intermediate phase **2fa′**, it was important to also record the initial transformation from **2fa** to **2fa′** in single-crystal to single-crystal fashion. Since the transformation from **2fa** to **2fa′** is a time consuming process at room temperature, gentle heating was used to bring about the conversion. Single-crystal diffraction data were collected for a suitable crystal of **2fa** at 100 K in order to indisputably verify the starting phase. The temperature was then ramped to 313 K to gently heat the crystal, followed by recollection of single-crystal diffraction data, which yielded a structure quite similar to that of **2fa′** isolated from the activated of bulk crystals (the only difference being that the structure still contains some residual solvent, owing to the much lower activation temperature). The single-crystal conversion of **2fa** to **2fa′** and subsequent conversion of **2fa′** to **3fa** explains the series of events involved during the transformation from a doubly- to a triply-interpenetrated structure. It is clear that the doubly-interpenetrated framework transforms into its triply-interpenetrated analog *via* an intermediate doubly-interpenetrated guest-free structure, which is severely distorted (Scheme 1) but distinct from **2fa**. Indeed, **2fa′** is the first intermediate structure isolated to date for such types of transformations, and it substantiates the mechanism that we proposed in our previous report. Although we also attempted to record the transformation from **2fa** to **2fa′** to **3fa** on the same crystal, it did not survive the process intact; taken together, our results imply that selecting a suitable crystal that would survive both transformations intact is a matter of chance. However, we were able to monitor the bulk change from **2fa** to **3fa** proceeding *via***2fa′** by using XRPD analysis of the same batch of crystals (Fig. S9†).

When gas sorption results are difficult to rationalize based on the as-synthesized host structure, it is generally assumed that the framework might have collapsed, thus resulting in less than anticipated surface area. The exact nature of this “collapse” is seldom investigated and the material is dismissed as unpromising. Given the often stated importance of developing new porous materials, it is surprising that little attention has been devoted to understanding the problems related to unexpected loss of porosity.

**Fig. 6:**
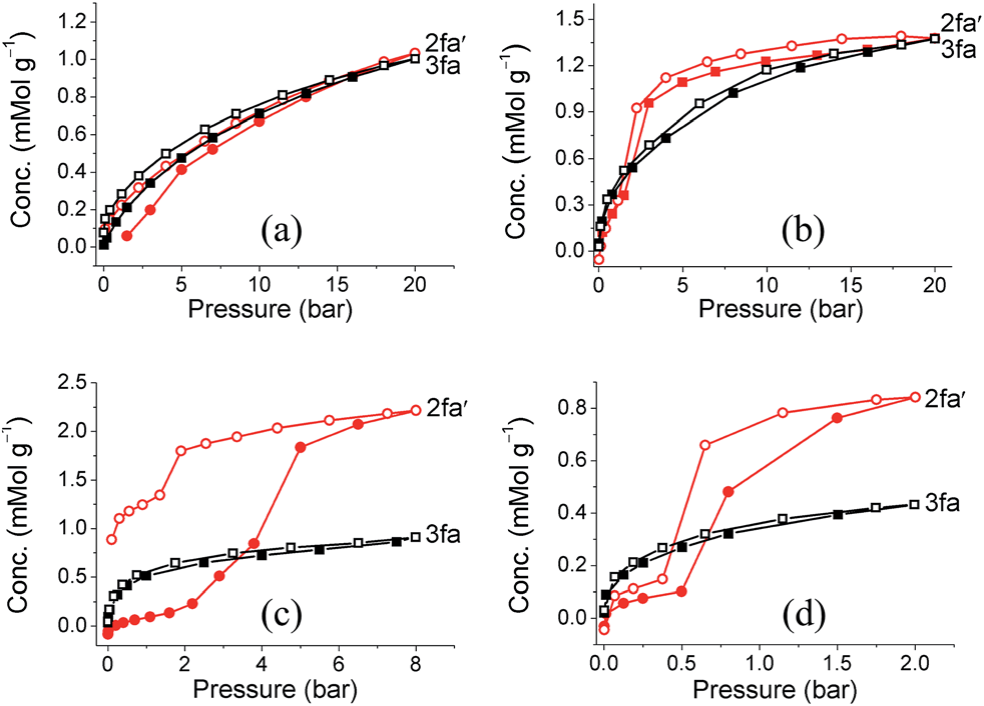
(a) Methane (b) ethane, (c) propane and (d) butane sorption isotherms of **2fa′** (circles) and **3fa** (squares) at 298 K (shaded and open symbols represent adsorption and desorption, respectively).

Since our first report on the change in degree of interpenetration of a MOF upon activation, we have maintained that this phenomenon might still be a relatively unappreciated reason for loss of porosity in similar materials. However, in both our previous reports^17^,^20^ we were not able to isolate the empty structure of the less interpenetrated form (the activated structure) and we could therefore not provide sufficient evidence to support our argument. Gas sorption studies were carried out on both guest-free phases **2fa′** and **3fa** in order to compare their sorption properties. PXRD patterns were recorded for **2fa′** before and after the gas sorption measurements to rule out the possibility of conversion of **2fa′** to **3fa** during the sorption experiment (Fig. S10†). Sorption isotherms were recorded at 298 K for carbon dioxide, methane, ethane, propane and butane (Fig. 6). Sorption of CO_2_ shows comparable gas uptake for both **2fa′** and **3fa** (Fig. S11†) and similar sorption trends were also observed for both phases in the case of methane. The similarity in gas uptake of CO_2_, and methane can be attributed to the comparable void volumes for both **2fa′** and **3fa** as determined using the Platon software package.

For the higher alkanes clear differences were observed for the sorption behavior of the two forms. Form **3fa** shows greater uptake of propane and butane at low pressure, which may be due to the catenation effect.^26^ However, in the case of **2fa′** there are clear steps between 2 to 5 bar for propane and 0.5 to 2 bar for butane, whereas no such steps are observed for **3fa**. Moreover, the propane and butane isotherms exhibit substantial hysteresis for **2fa′**. This phenomenon can be related to the so-called “gate opening effect”, which is quite common for flexible/dynamic MOFs.^27^ Indeed, it is interesting to note that **2fa′** shows greater uptake of these gases at higher pressure, which suggests enhancement of porosity, possibly due to gate opening. With regard to the sorption isotherms for ethane, a step is also apparent in the case of **2fa′**, albeit less pronounced than for propane or butane. However, for both **2fa′** and **3fa** the extent of hysteresis for ethane sorption is relatively insignificant and the total uptake at 20 bar is approximately the same.

## Conclusions

The isolation and characterization of structural intermediates is crucial for understanding structure–-property relationships in dynamic MOFs, and it also extends our knowledge of concepts such as permanent and transient porosity. The [Co_2_(ndc)_2_(bpy)] system reported by Chen and co-workers was presumed to lose porosity upon activation, leading to lower than anticipated surface area for such a seemingly highly porous framework.^14^ We believe that the activated framework isolated by Chen *et al.* was **3fa** and not **2fa′** (owing to activation at high temperature in their case). This highlights the notion that exact activation conditions can be critical for gas sorption studies. Although much attention is usually devoted to design criteria and crystallization methodologies, activation procedures receive comparatively little consideration. The control of interpenetration in MOFs has also received much attention and it is generally expected that once a less interpenetrated structure is isolated, it will retain its interconnectivity upon activation. In the present case the doubly-interpenetrated framework of [Co_2_(ndc)_2_(bpy)] has indeed been produced by altering the cystallization conditions but upon activation at 120 °C it converts to its triply-interpenetrated form. However, under milder activation conditions the same material yields the empty twofold-interpenetrated structure. We also show that misleading results might be obtained if a sample is kept under ambient conditions for some time before the sorption data are recorded. To date the phenomenon of a change in degree of interpenetration and its effect on porosity has largely been overlooked. This study demonstrates the importance of considering dramatic structural rearrangements when rationalizing unexpected loss of porosity. We believe that the phenomenon needs more attention and that many more systems (both known and unknown) might experience similar changes. Indeed, these changes may go unnoticed owing to the difficulties involved in analyzing materials that lose crystal singularity upon activation.

## Supplementary Material

Supplementary information

Crystal structure data
