# Supplementary material for: Isolation of a structural intermediate during switching of degree of interpenetration in a metal–organic framework
Source: Chem Sci. 2015 Jun 11;6(8):4986–92. doi: 10.1039/c5sc01796c (PMC6088436; doi:10.1039/c5sc01796c)
Supplement: Supplementary file 1 [file SC-006-C5SC01796C-s001.pdf]

Electronic Supplementary Material (ESI) for Chemical Science.
This journal is © The Royal Society of Chemistry 2015

Supporting Information

Isolation of a Structural Intermediate During Switching of Degree of Interpenetration in a Metal-Organic Framework.

*Himanshu Aggarwal, Raj Kumar Das, Prashant M. Bhatt and Leonard J. Barbour**

Department of Chemistry and Polymer Science, University of Stellenbosch, Matieland 7602, Stellenbosch, South Africa

EXPERIMENTAL

Methods and materials. All chemicals were obtained commercially and used as received without further purification.

Synthesis of 2fa. The doubly-interpenetrated $[Co_2(ndc)_2(bpy)]$ metal-organic framework was synthesized following a previously reported procedure.[1]

Preparation of 3fa by activation of 2fa. Crystals of the triply-interpenetrated structure **3fa** were prepared by activating **2fa** crystals under dynamic vacuum at 120 °C for 12 hours in a Büchi glass oven. Crystals of **3fa** were also obtained when **2fa** crystals were removed from mother liquor and left standing under ambient conditions for approximately two weeks.

Preparation of 2fa′ by activation of 2fa. Crystals of the empty doubly interpenetrated structure **2fa′** were prepared by activating **2fa** crystals under dynamic vacuum at 80 °C for 12 hours in a Büchi glass oven.

Single-crystal X-ray diffraction (SCD). Intensity data were collected on a Bruker Apex II DUO CCD diffractometer with a multilayer monochromator. Mo-K_α radiation (λ= 0.71073 Å) was used for the experiments. The temperature of the crystal was controlled using an Oxford Cryostream 700 Plus. Data reduction was carried out by means of standard procedures using the Bruker software package SAINT[2] while absorption corrections and correction of other systematic errors were carried out using SADABS.[3] The structures were solved by direct methods using SHELXS-97 and refined using SHELXL-97.[4] X-Seed[5] was used as the graphical interface for the SHELX program suite. Hydrogen atoms were placed in calculated positions using riding models. In the case of **2fa** the bpy linker is disordered over three positions (Figure S12). As a result, the thermal parameters of the bpy fragment are substantially different, resulting in some A and B level alerts in the checkcif report. Moreover, one of the nitrogen atoms (N2A) was refined isotropically. The disorder was

modelled to the best of our ability to make sense of the electron density maps. The remaining electron density within the channel was removed using the SQUEEZE routine of PLATON[24]. The solvent-accessible void volumes were also calculated using PLATON.

Single-crystal to single-crystal conversion of 2fa to 3fa. A single crystal of **2fa** was left at room temperature. The unit cell parameters for the same crystal were checked at regular intervals to monitor the progress of the transformation. Based on these parameters, the conversion appeared to be complete after almost one week and the single-crystal X-ray diffraction data analysis revealed that the system had converted to its triply-interpenetrated form (**3fa**).

Single-crystal to single-crystal conversion of 2fa′ to 3fa. A suitable single crystal of **2fa′** isolated from the bulk material was left at room temperature. The unit cell parameters of the same crystal were checked at regular intervals to monitor the progress of the transformation. After 3 to 4 days the conversion appeared to be complete and single-crystal X-ray diffraction analysis revealed that the system had converted to its triply-interpenetrated form (**3fa**).

Single-crystal to single-crystal conversion of 2fa to 2fa′. Since the transformation from **2fa** to **2fa′** is a time consuming process at room temperature, gentle heating was used to bring about the conversion. A suitable crystal of as-synthesized material was glued to the end of a glass fibre and SCD data were recorded at 100 K, yielding the structure of **2fa**. The temperature was then ramped to 313 K at a rate of 120 K h^{-1} and kept constant while unit cell parameters were determined repeatedly. Once the unit cell parameters indicated apparentlycomplete conversion of **2fa** to **2fa′**, the temperature was then decreased back to 100 K. Single crystal X-ray diffraction data were again recorded, revealing a structure quite similar to that of **2fa′** isolated from the activation of bulk crystals.

Thermogravimetric analysis. Thermal analyses were carried out using a TA Instruments Q500 thermogravimetric analyser.

X-ray powder diffraction studies (XRPD). The diffractograms were recorded with a Bruker D2 PHASER equipped with a Lynxeye 1D detector and Ni-filtered copper Kα radiation (30 kV, 10 mA generator parameters; restricted by a 1.0 mm divergence slit and a 2.5° Soller collimator) with a 0.02° step width. The phase purity of the as-synthesized **2fa** and the activated forms **2fa′** and **3fa** was confirmed by comparison of experimental XRPD with the simulated XRPD patterns from the respective crystal structures. XRPD was used to study all the conversions under different conditions.

Gas sorption measurement. An Intelligent Gravimetric Analyser (IGA-002 supplied by Hiden Analytical Ltd, Warrington, UK)[6] was used to record sorption isotherms at 298 K for samples of **2fa′** and **3fa**. The instrument facilitates precise measurement of mass change and control of pressure and temperature, and is equipped with an advanced pressure rating allowing measurement up to 20 bar. The pressure is monitored using a pressure transducer with a range of 0-20 bar and buoyancy effects are corrected for. Temperature control is maintained to an accuracy of ca. 0.05 °C by a refrigerated recirculating bath. Data collection is controlled by Real-Time Processing computer software,[7] which continually analyses the equilibrium using least-squares regression to extrapolate a value of the asymptote.

Single-crystal X-ray diffraction (SCD)

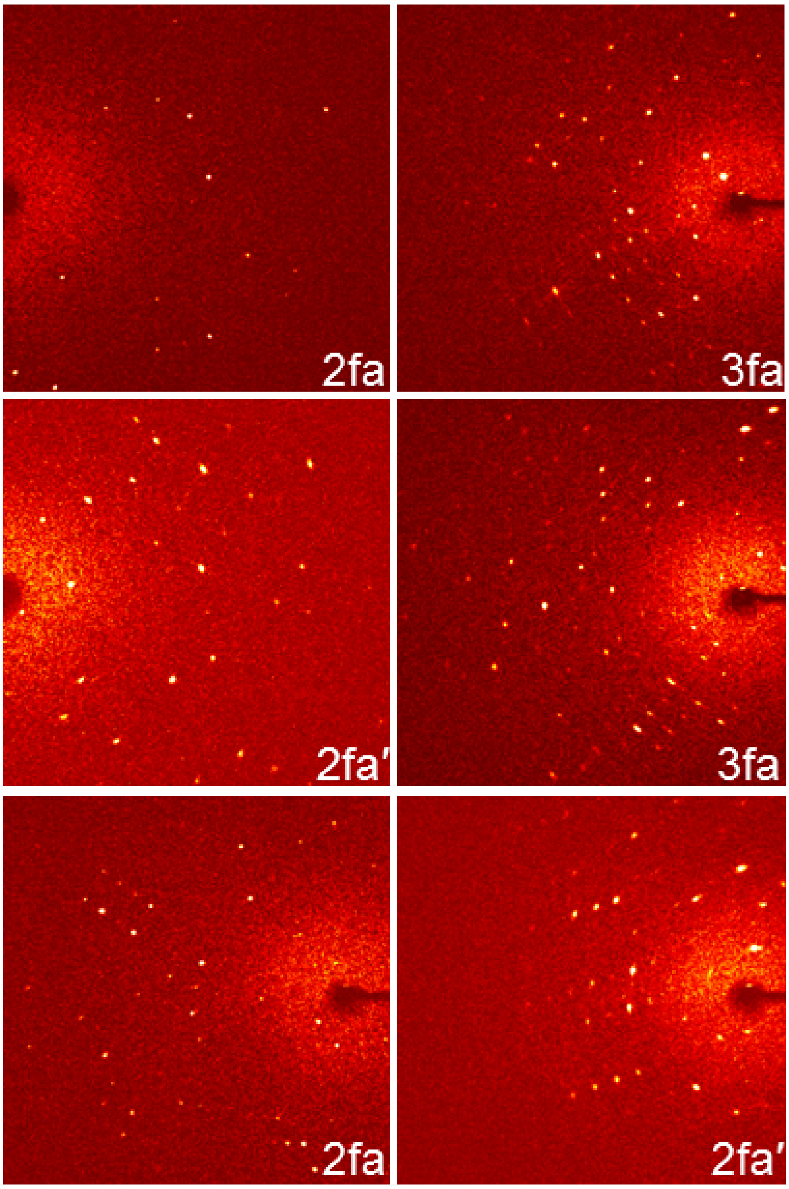

Diffraction patterns for three single-crystal to single-crystal transformations: **2fa to 3fa** (top), **2fa′ to 3fa** (middle) and **2fa to 2fa′** (bottom).

Crystal Data and Structure Refinement:

Identification code	**LJB_2fa_1**	**LJB_3fa_1**
Empirical formula	$C_{34}H_{20}Co_2N_2O_8$	$C_{51}H_{30}Co_3N_3O_{12}$
Formula weight	702.38	1053.57
Temperature/K	100(2)	296(2)
Crystal system	triclinic	monoclinic
Space group	*P*-1	*C*2
a/Å	12.956(3)	17.173(6)
b/Å	13.122(3)	19.784(7)
c/Å	13.752(3)	13.887(5)
α/°	84.973(3)	90.00
β/°	67.595(3)	95.771(4)
γ/°	83.245(3)	90.00
Volume/$Å^3$	2144.0(8)	4694(3)
Z	2	4
$\rho_{calc}g/cm^3$	1.088	1.491
μ/mm^{-1}	0.814	1.115
F(000)	712.0	2136.0
Crystal size/mm^3	0.2 × 0.08 × 0.06	0.24 × 0.14 × 0.04
Radiation	MoKα (λ = 0.71073)	MoKα (λ = 0.71073)
2θ range for data collection/°	3.2 to 52.12	2.94 to 56.76
Index ranges	$-16 \leq h \leq 16$, $-16 \leq k \leq 16$, $-16 \leq l \leq 16$	$-22 \leq h \leq 22$, $-26 \leq k \leq 26$, $-18 \leq l \leq 18$
Reflections collected	21004	56788
Independent reflections	8409 [R_{int} = 0.0506, R_{sigma} = 0.0733]	11722 [R_{int} = 0.0710, R_{sigma} = 0.0615]
Data/restraints/parameters	8409/42/392	11722/1/467
Goodness-of-fit on F^2	1.038	1.052
Final R indexes [I>=2σ (I)]	R_1 = 0.0534, wR_2 = 0.1270	R_1 = 0.0615, wR_2 = 0.1659
Final R indexes [all data]	R_1 = 0.0801, wR_2 = 0.1375	R_1 = 0.0938, wR_2 = 0.1840
Largest diff. peak/hole / e $Å^{-3}$	0.85/-1.29	1.16/-1.11
Mosaicity	0.35	0.40
**CCDC Number**	1052168	1052172

Identification code	**LJB_2fa'_1**	**LJB_3fa_2**
Empirical formula	$C_{17}H_{10}CoNO_4$	$C_{51}H_{30}Co_3N_3O_{12}$
Formula weight	351.19	1053.57
Temperature/K	296(2)	100(2)
Crystal system	triclinic	monoclinic
Space group	*P*-1	*C*2
a/Å	7.7116(7)	17.0391(8)
b/Å	10.2889(10)	20.0278(10)
c/Å	11.0085(10)	13.8334(7)
α/°	71.4300(10)	90.00
β/°	86.2820(10)	96.243(3)
γ/°	82.2650(10)	90.00
Volume/Å^3	820.22(13)	4692.7(4)
Z	2	4
ρ_{calc}g/cm^3	1.422	1.491
μ/mm^{-1}	1.063	1.115
F(000)	356.0	2136.0
Crystal size/mm^3	0.12 × 0.05 × 0.02	0.13 × 0.05 × 0.02
Radiation	MoKα (λ = 0.71073)	MoKα (λ = 0.71073)
2θ range for data collection/°	3.9 to 56.6	2.96 to 56.88
Index ranges	-10 ≤ h ≤ 10, -13 ≤ k ≤ 13, -14 ≤ l ≤ 14	-22 ≤ h ≤ 22, -26 ≤ k ≤ 26, -18 ≤ l ≤ 18
Reflections collected	23571	43655
Independent reflections	4064 [R_{int} = 0.0574, R_{sigma} = 0.0421]	11692 [R_{int} = 0.0883, R_{sigma} = 0.0893]
Data/restraints/parameters	4064/24/236	11692/46/443
Goodness-of-fit on F^2	1.062	1.026
Final R indexes [I>=2σ (I)]	R_1 = 0.0492, wR_2 = 0.1217	R_1 = 0.0588, wR_2 = 0.1373
Final R indexes [all data]	R_1 = 0.0720, wR_2 = 0.1331	R_1 = 0.1141, wR_2 = 0.1654
Largest diff. peak/hole / e Å^{-3}	1.60/-0.53	1.44/-1.42
Mosaicity	0.41	0.40
**CCDC Number**	1052169	1052173

Identification code	**LJB_2fa_2**	**LJB_2fa'_2**
Empirical formula	$C_{34}H_{20}Co_2N_2O_8$	$C_{17}H_{10}CoNO_4$
Formula weight	702.38	351.19
Temperature/K	100(2)	100(2)
Crystal system	triclinic	triclinic
Space group	*P*-1	*P*-1
a/Å	12.9698(12)	7.6397(12)
b/Å	13.1304(12)	10.4311(17)
c/Å	13.7579(13)	10.9968(18)
α/°	85.0410(10)	71.121(12)
β/°	68.0140(10)	87.061(12)
γ/°	83.544(2)	83.361(12)
Volume/Å^3	2156.3(3)	823.5(2)
Z	2	2
ρ_{calc}g/cm^3	1.082	1.416
μ/mm^{-1}	0.809	1.059
F(000)	712.0	356.0
Crystal size/mm^3	0.2 × 0.12 × 0.04	0.19 × 0.11 × 0.05
Radiation	MoKα (λ = 0.71073)	MoKα (λ = 0.71073)
2θ range for data collection/°	3.12 to 56.72	3.92 to 50.32
Index ranges	$-17 \leq h \leq 17$,<br>$-17 \leq k \leq 17$,<br>$-18 \leq l \leq 18$	$-9 \leq h \leq 9$,<br>$-12 \leq k \leq 12$,<br>$-13 \leq l \leq 13$
Reflections collected	54070	8403
Independent reflections	10710 [R_{int} = 0.0381,<br>R_{sigma} = 0.0303]	2879 [R_{int} = 0.0861,<br>R_{sigma} = 0.1013]
Data/restraints/parameters	10710/20/458	2879/6/251
Goodness-of-fit on F^2	1.096	1.087
Final R indexes [I>=2σ (I)]	R_1 = 0.0391,<br>wR_2 = 0.0989	R_1 = 0.0958,<br>wR_2 = 0.2632
Final R indexes [all data]	R_1 = 0.0487,<br>wR_2 = 0.1023	R_1 = 0.1276,<br>wR_2 = 0.2806
Largest diff. peak/hole / e Å^{-3}	1.26/-1.07	1.28/-0.72
Mosaicity	0.40	0.40
**CCDC Number**	1052170	1052171

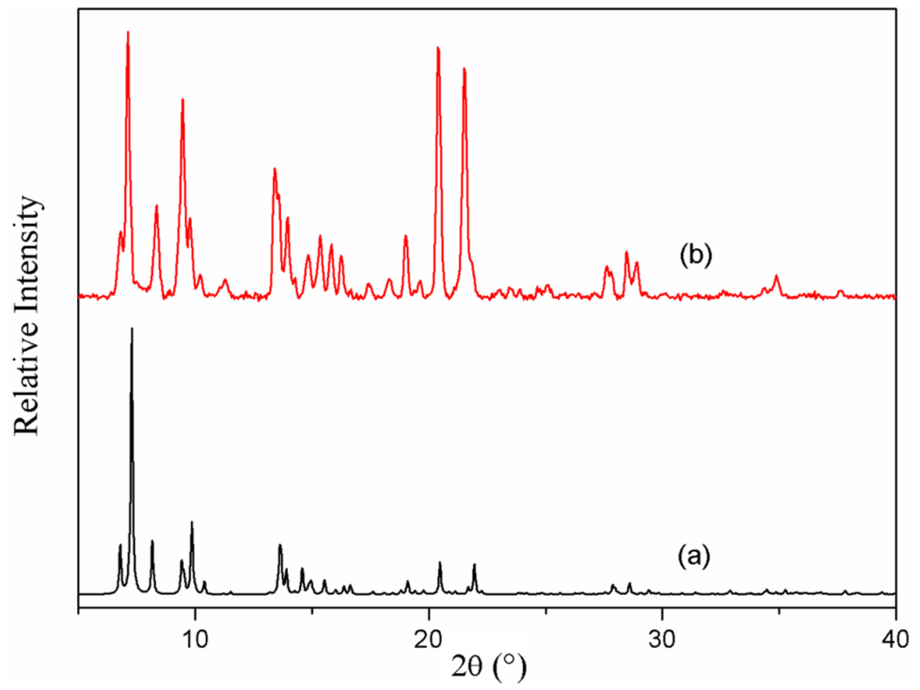

Fig. S1: (a) simulated XRPD pattern of **2fa** from crystal structure, (b) XRPD patterns of as-synthesized **2fa**.

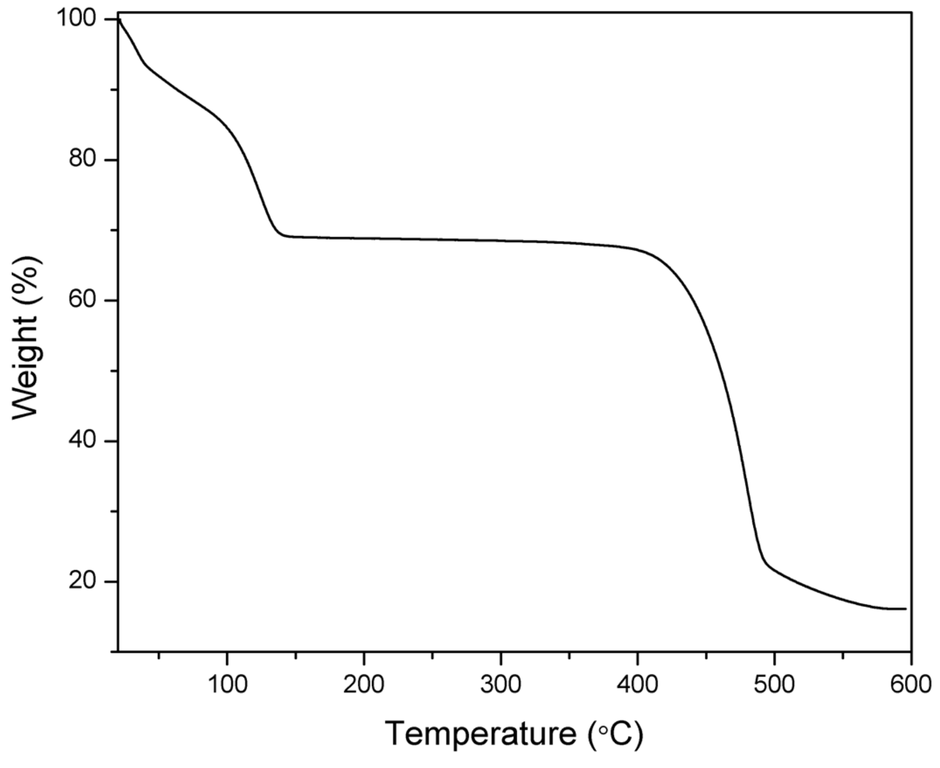

Fig. S2: TGA analysis of the as-synthesized crystals of **2f**

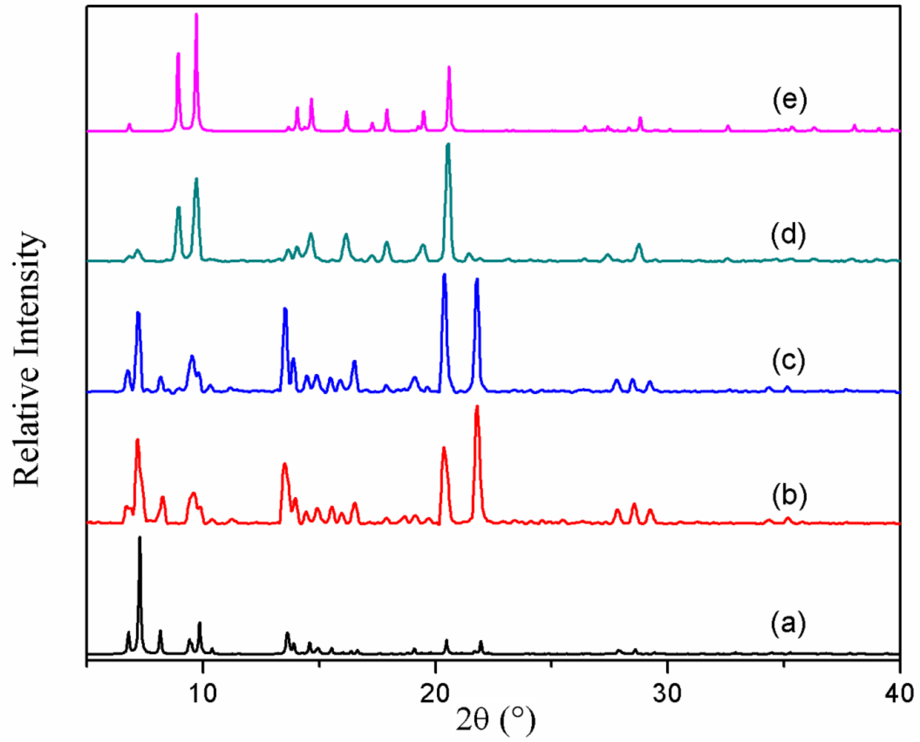

Fig. S3: (a) Simulated XRPD pattern of **2fa** from crystal structure, (b) XRPD pattern of as-synthesized **2fa**. (c) XRPD of the **2fa** sample after on exposure to ambient conditions for 5 days, (d) XRPD of the **2fa** sample after exposure to ambient condition for 10 days (e) simulated XRPD pattern of **3fa** from crystal structure.

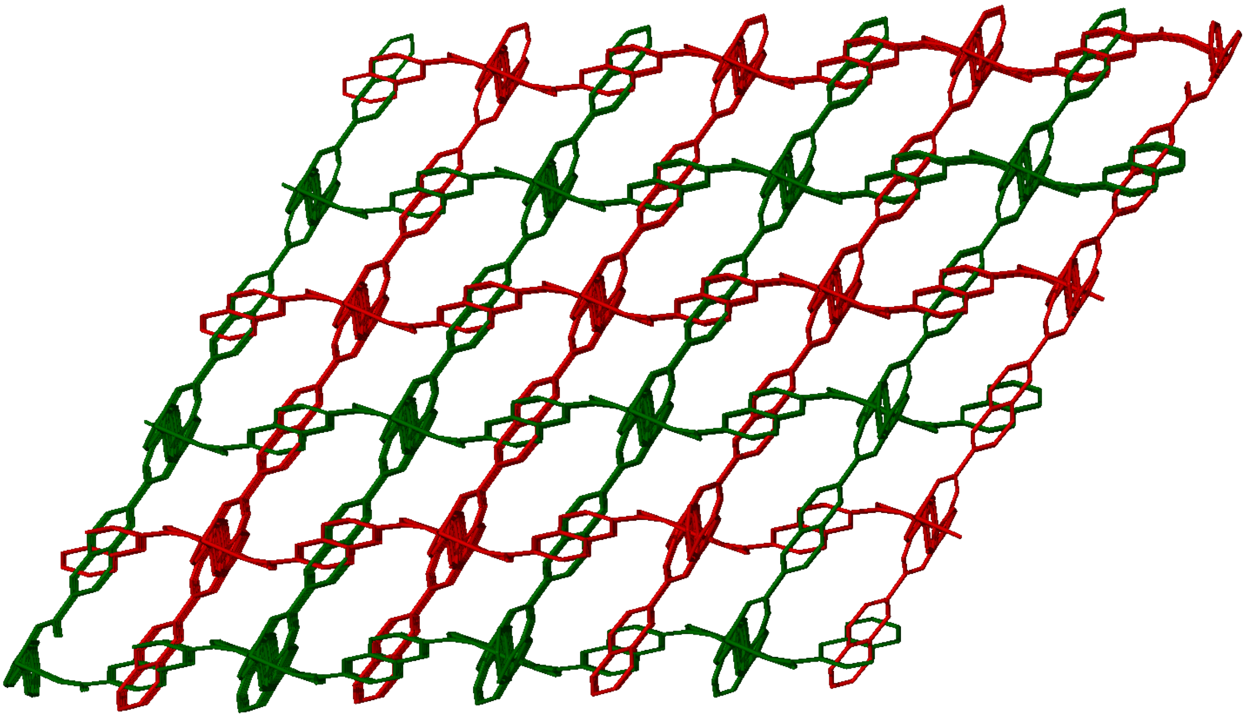

Fig. S4: Packing diagram of **2fa′** (H atoms have been removed for clarity)

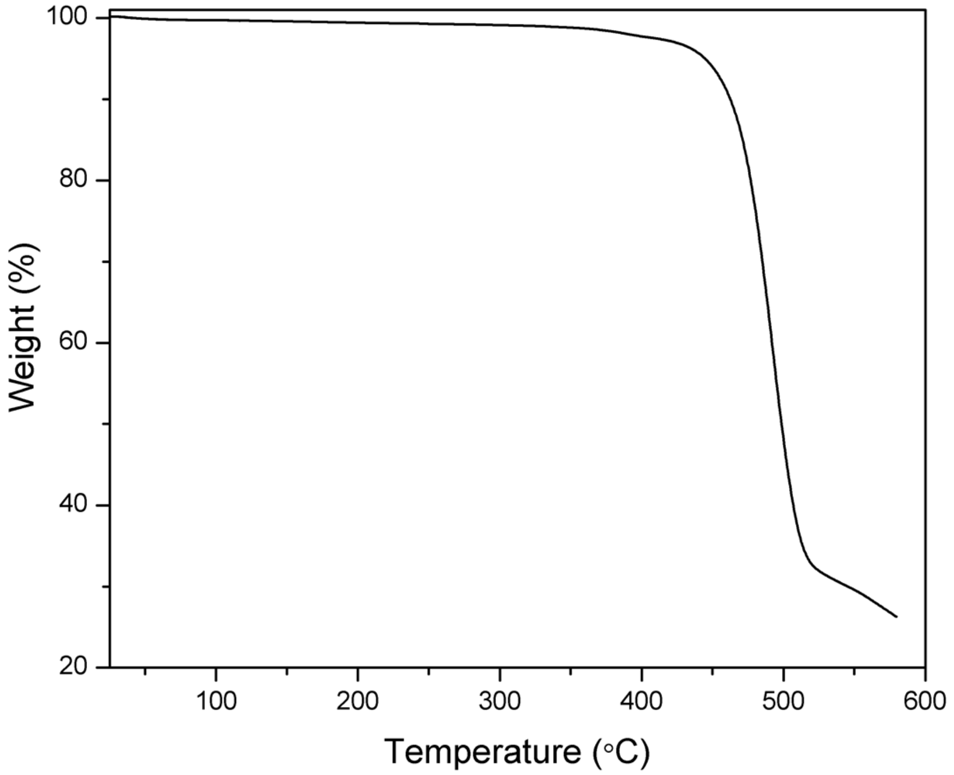

Fig. S5: TGA analysis of **2fa′** sample.

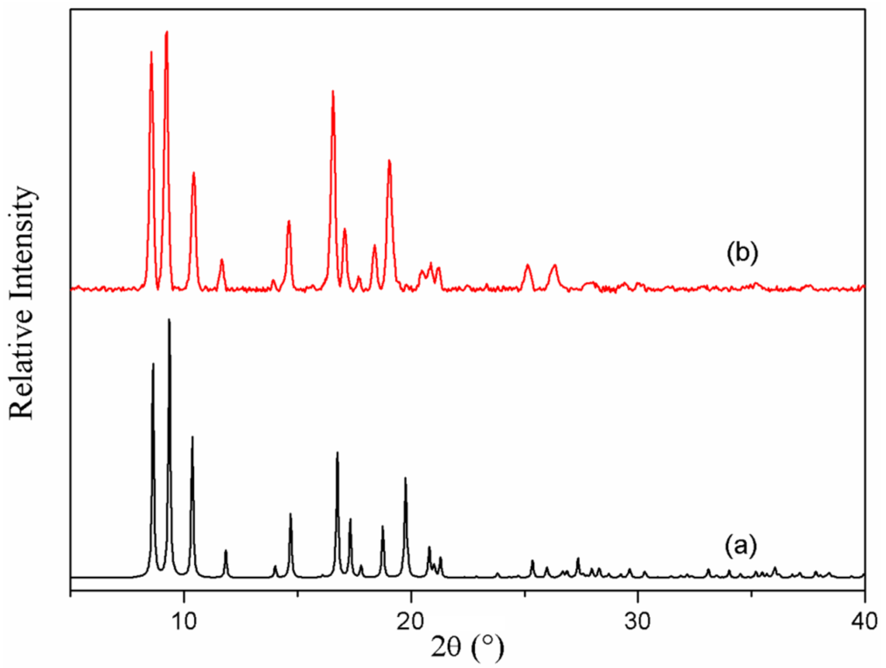

Fig. S6: (a) simulated XRPD pattern of **2fa′** from crystal structure, (b) XRPD patterns of as-synthesized **2fa′**.

Table S1: Comparison of Co-O and Co-N bond distances in 2fa and 2fa′

2fa		2fa′	
Co1—O5	2.012(1)	Co1—O4^{i}	2.007(3)
Co1—O7^{i}	2.025(1)	Co1—O3	2.032(3)
Co1—O3^{ii}	2.029(2)	Co1—O2^{i}	2.058(2)
Co1—O1	2.097(2)	Co1—O1	2.176(3)
Co1—Co2	2.764(1)		
O3—Co1^{iv}	2.029(2)		
O4—Co2^{iv}	2.080(2)		
O7—Co1^{v}	2.025(1)		
O8—Co2^{v}	2.020(1)		
Co2—O8^{i}	2.020(1)		
Co2—O6	2.023(1)		
Co2—O2	2.025(2)		
Co2—O4^{ii}	2.080(2)		
**Average Co—O**	**2.039(1)**	**Average Co—O**	**2.068(4)**
Co2—N1A	2.055(7)	Co1—N1A	2.056(20)
Co2—N1C	2.016(13)	Co1—N1B	2.077(18)
Co2—N1B	2.104(15)		
Co1—N2A^{iii}	2.068(4)		
Co1—N2B^{iii}	2.070(18)		
Co1—N2C^{iii}	2.091(19)		
**Average Co—N**	**2.067(5)**	**Average Co—N**	**2.067(13)**
(i) x, -1+y, z; (ii) -1+x, y, z; (iii) x, y, 1+z; (iv) 1+x, y, z; (v) x, 1+y, z; (vi) x, y, -1+z.		(i) 1-x, 1-y, 1-z; (ii) 1-x, 2-y, -z; (iii) -x, 1-y, 2-z; (iv) 2-x, 2-y, 1-z.	

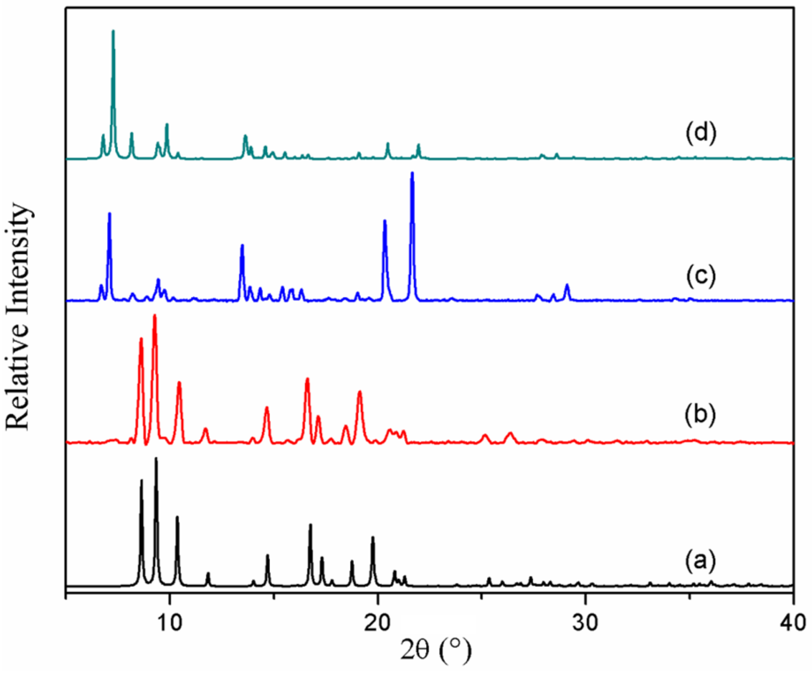

Fig. S7: (a) Simulated XRPD pattern of **2fa′** from the SCD structure, (b) XRPD pattern of bulk **2fa′**, (c) XRPD pattern of **2fa′** after immersion in fresh DMF at RT for 24 hours and (d) simulated XRPD pattern of **2fa** from SCD data.

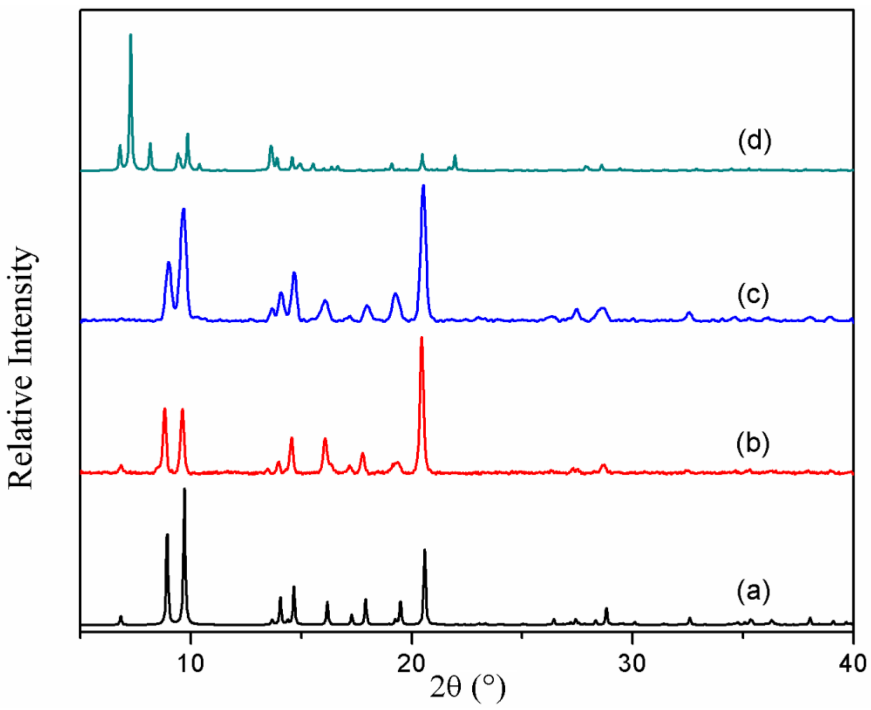

Fig. S8: a) Simulated XRPD pattern of **3fa** from the SCD structure, (b) XRPD pattern of bulk **3fa**, (c) XRPD pattern of **3fa** after immersion in fresh DMF at 120 °C for 48 hours and (d) simulated XRPD pattern of **2fa** from SCD data.

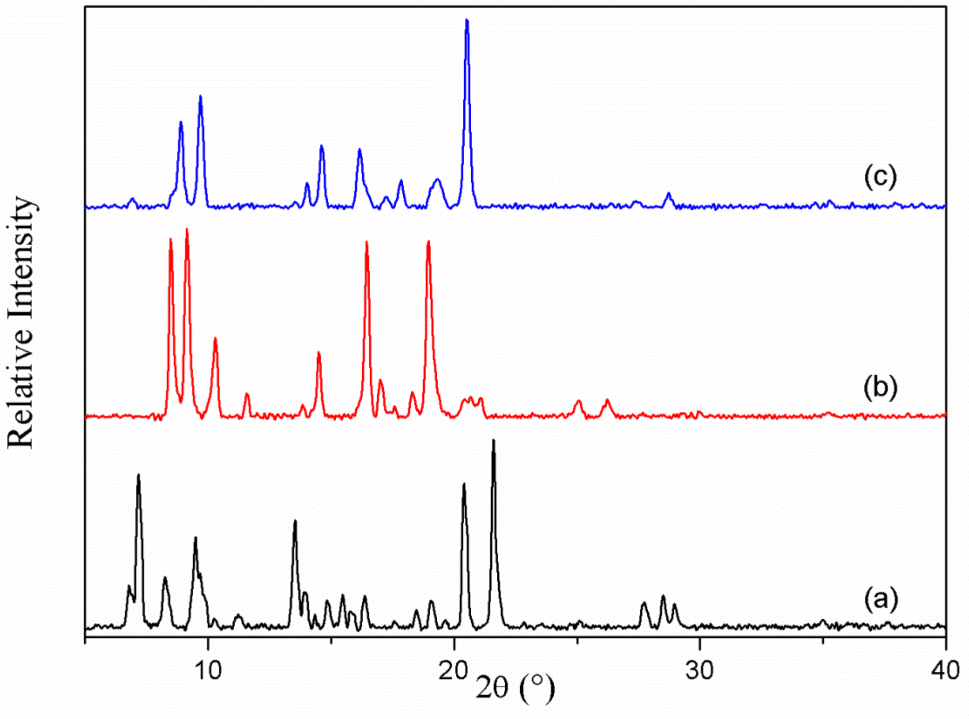

Fig. S9: (a) XRPD pattern of as-synthesized **2fa**. (b) XRPD patterns of the sample after heating at 80 °C under dynamic vacuum, (c) XRPD pattern of 80 °C activated sample after further activation at 120 °C under dynamic vacuum. **(These XRPD patterns clearly show subsequent conversion from 2fa to 2fa′ to 3fa)**

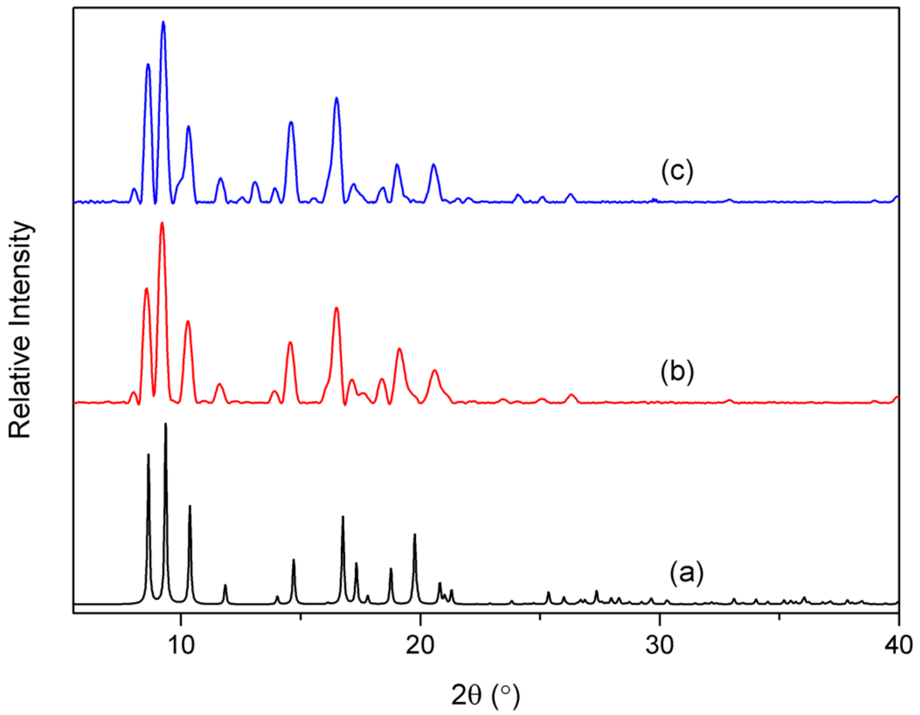

Fig. S10: (a) Simulated XRPD pattern of **2fa′** from the SCD structure, (b) XRPD pattern of bulk **2fa′** before sorption experiment, (c) XRPD pattern of bulk **2fa′** after sorption experiment.

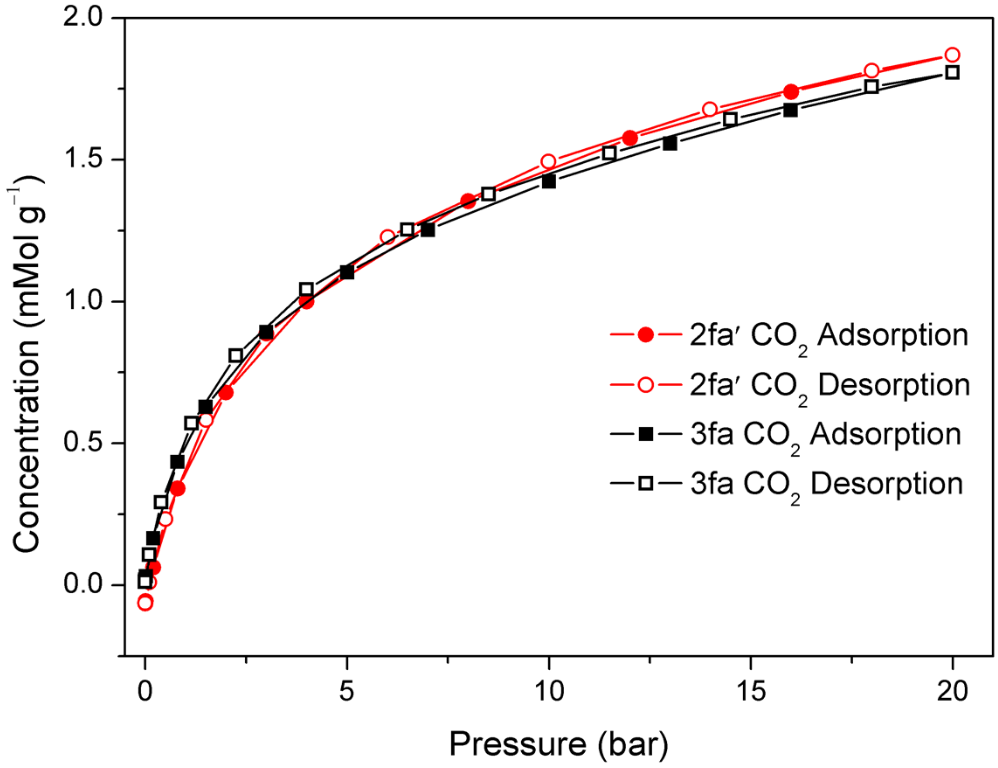

Fig. S11: CO_2 sorption isotherm of **2fa′**and **3fa** at 298K.

Measurement of dihedral and torsion angles:

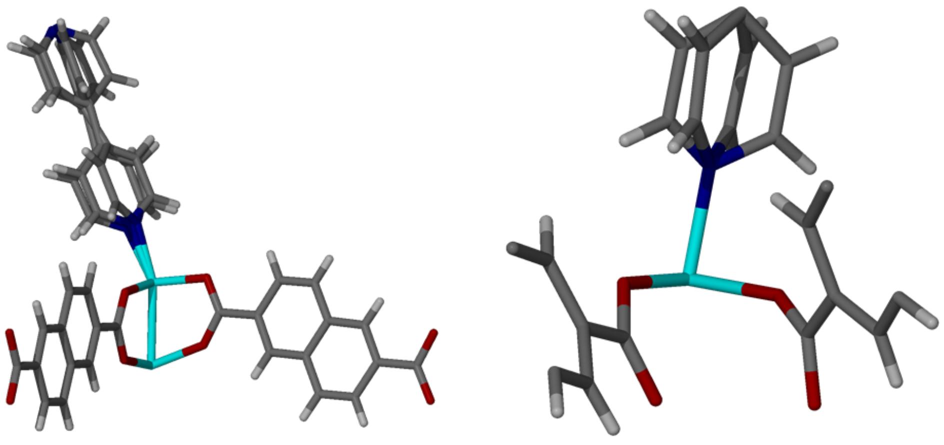

2fa **2fa′**

Fig. S12: Asymmetric units of 2fa and 2fa′

The dihedral angles between metal plane and metal-carboxylate plane (θ) as well as metal plane and the aromatic plane of ndc (φ) in both **2fa** and **2fa′** structures were measured. There are two crystallographically distinct ndc linkers with four different carboxylates and two different aromatic units in **2fa**. As a result, a total of four θ and two φ values were determined. In the case of the bpy linker, similar measurements were made by measuring the torsion angle (δ) between the metal-metal axis and the metal-N linkages and torsion angle (ω) between metal-metal axis and aromatic part of bpy. Since bpy is disordered over three positions in **2fa** (as shown above), torsion angles for each individual subunit were measured and the complete range reported. Similar meassurement were carried out for **2fa′**.

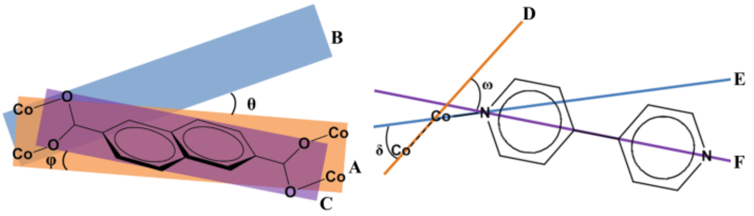

2fa				2fa'			
θ	φ	δ	ω	θ	φ	δ	ω
1.56(4)	2.98(5)	17.8(1) – 29.5(3)	21.7(1) – 27.46(4)	10.53(3)	7.41(6)	30.1(3) – 35.3(3)	33.7(1) – 36.8(1)
1.71(4)	7.81(4)	20.8(7) – 28.9(3)		18.63(4)	18.33(4)		
3.70(3)							
4.44(4)							

Top: diagrammatic representation of key dihedral and torsion angles in **2fa** and **2fa'**. A: $Co_2 \cdots Co_2$ metal atom plane; B: Co_2-carboxylate oxygen plane; C: aromatic plane of ndc; D: Co_2 cluster metal-metal axis; E: metal-nitrogen; F: N···N axis of bpy. Both crystallographic symmetry and geometrical disorders have been considered in measuring these parameters. **Bottom**: table of relevant angles in º.

Measurement of metal-metal distances between two paddlewheels:

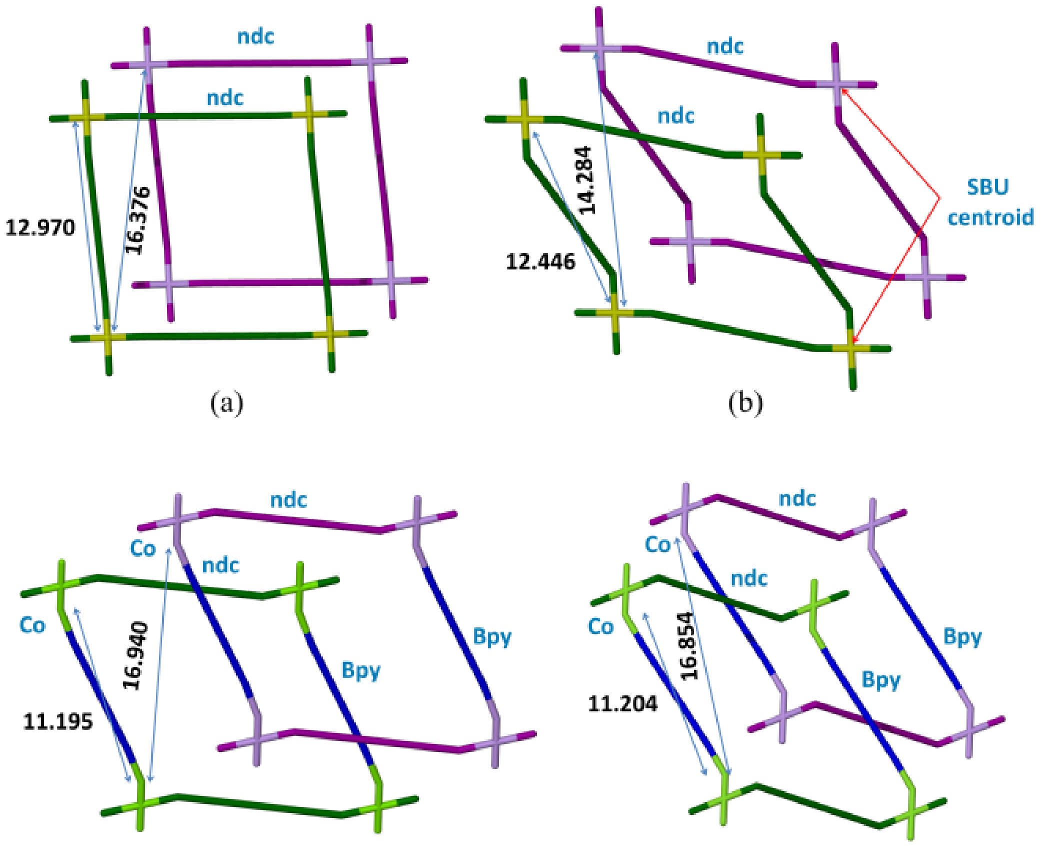

(a) (b) (c) (d)

In the case of ndc, the node···node distances between two paddlewheel units is taken as the distance between the Co–Co centroids of the two metal clusters bridged by the ndc ligand. The same protocol was used to determine the shortest distance between two paddlewheel nodes of different networks. In the case of bpy, the distance is measured between the two Co metal centers to which the ligand is attached. It should be noted that only the geometrically favourable Co centers are taken in to account while measuring the shortest distances between paddlewheel units of different networks.

Rietveld Refinement

The observed X-ray powder patterns were refined using the Rietveld[8] method. The program TOPAS was employed, using the corresponding published single-crystal X-ray structures as starting models. The resultant difference plots thus generated are given below.

NOTE:

For Rietveld refinements, the background was not subtracted for any of the three XRPD patterns. The XRPD patterns were originally collected on Bruker D2 phaser equipped with a Cu source. Owing to the presence of Co ions, each pattern contained a significant background signal, which was subtracted (for clarity) when comparing the XRPD patterns with simulated patterns obtained from single-crystal data.

Table S3 Final Rietveld refinement parameters for the three structures:

Compound	**2fa**	**2fa′**	**3fa**
$R_p$fitted	0.010	0.008	0.018
$_wR_p$fitted	0.014	0.011	0.024
Bragg R-factor	2.073	1.403	0.286
GoF (χ)	2.04	1.64	1.21
Temperature (K)	298(2)	298(2)	298(2)
Space group	*P-1*	*P-1*	*C2*
a (Å)	12.9377(46)	7.7014(47)	17.2570(74)
b (Å)	13.0741(27)	10.2658(63)	19.832(12)
c (Å)	13.7277(62)	10.9889(87)	13.9305(46)
α, (°)	85.656(54)	71.535(59)	90
β (°)	67.731(66)	86.330(52)	95.586(37)
γ (°)	84.071(55)	82.285(38)	90

Table S4: Comparison of Rietveld parameters with the single crystal unit cell parameters for 2fa

Compound	2fa	
	XRPD (Rietveld)	SCD (SHELXL)
Temperature (K)	298	100
Space group	*P-1*	*P-1*
a (Å)	12.9377(46)	12.9698(12)
b (Å)	13.0741(27)	13.1304(12)
c (Å)	13.7277(62)	13.7579(13)
α, (°)	85.656(54)	85.0410(10)
β (°)	67.731(66)	68.0140(10)
γ (°)	84.071(55)	83.544(2)

Table S5: Comparison of Rietveld parameters with the single crystal unit cell parameters for 2fa′

Compound	2fa′	
	XRPD (Rietveld)	SCD (SHELXL)
Temperature (K)	298	296
Space group	*P-1*	*P-1*
a (Å)	7.7014(47)	7.7116(7)
b (Å)	10.2658(63)	10.2889(10)
c (Å)	10.9889(87)	11.0085(10)
α, (°)	71.535(59)	71.4300(10)
β (°)	86.330(52)	86.2820(10)
γ (°)	82.285(38)	82.2650(10)

Table S5: Comparison of Rietveld parameters with the single crystal unit cell parameters for 3fa.

Compound	3fa	
	XRPD (Rietveld)	SCD (SHELXL)
Temperature (K)	298	296
Space group	*C2*	*C2*
a (Å)	17.2570(74)	17.173(6)
b (Å)	19.832(12)	19.784(7)
c (Å)	13.9305(46)	13.887(5)
α, (°)	90	90
β (°)	95.586(37)	95.771(4)
γ (°)	90	90

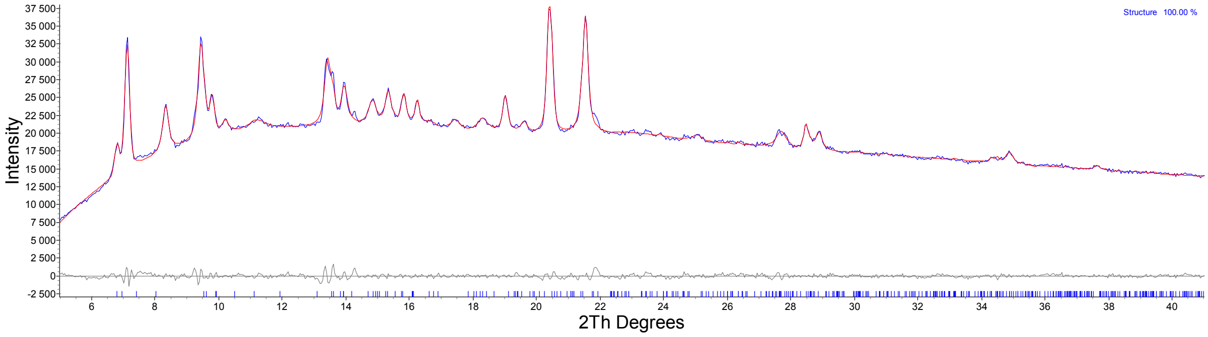

Fig. S13: Observed (blue) and simulated (red) X-ray powder diffractograms (the latter obtained from Rietveld refinement) as well as the difference plot (grey) for **2fa**.

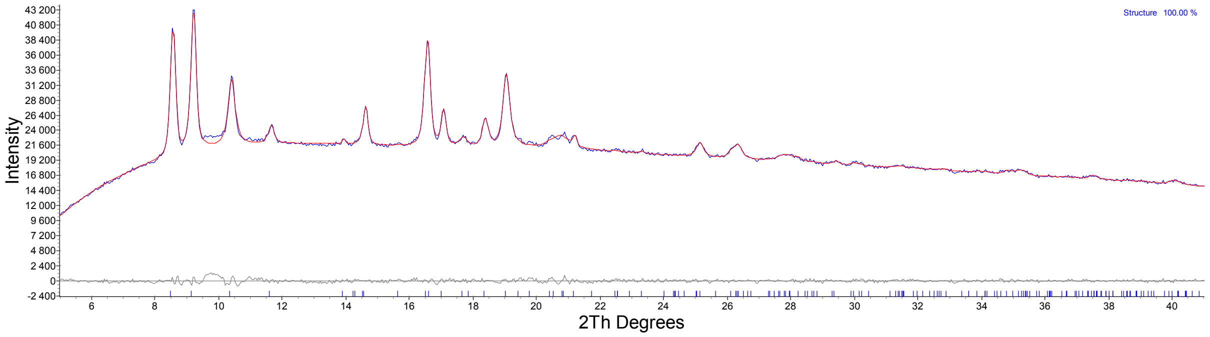

Fig. S14: Observed (blue) and simulated (red) X-ray powder diffractograms (the latter obtained from Rietveld refinement) as well as the difference plot (grey) for **2fa′**.

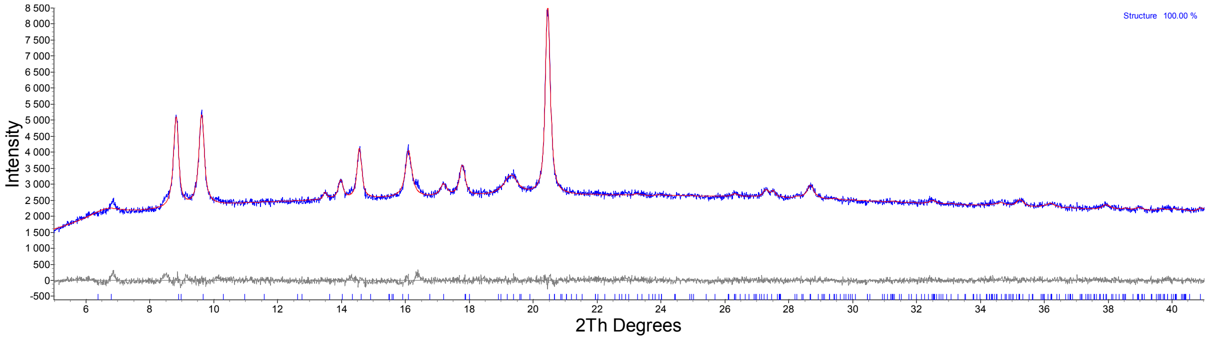

Fig. S15: Observed (blue) and simulated (red) X-ray powder diffractograms (the latter obtained from Rietveld refinement) as well as the difference plot (grey) for **3fa**.

References

1. B. Chen, S. Ma, E. J. Hurtado, E. B. Lobkovsky, C. Liang, H. Zhu and S. Dai, *Inorg. Chem.,* 2007, **46**, 8705.
2. SAINT Data Reduction Software, Version 6.45; Bruker AXS Inc., Madison, WI, 2003.
3. (a) SADABS, Version 2.05; Bruker AXS Inc., Madison, WI, 2002; (b) R. H. Blessing, *Acta Crystallogr., Sect. A: Found. Crystallogr.*, 1995, **51**, 33138.
4. G. M. Sheldrick, *Acta Crystallogr., Sect. A: Found. Crystallogr.*, 2008, **64**, 1121122.
5. L. J. Barbour, *J. Supramol. Chem.*, 2001, **1**, 189.
6. M. J. Benham and D. K. Ross, Z. Phys. Chem., 1989, **163**, 25.
7. (a) C. R. Reid, I. P. O'Koye and K. M. Thomas, *Langmuir*, 1998, **14**, 2415; (b) C. R. Reid and K. M. Thomas, *Langmuir*, 1999, **15**, 3206; (c) A. W. Harding, N. J. Foley, P.

R. Norman, D. C. Francis and K. M. Thomas, *Langmuir*, 1998, **14**, 3858; (d) I. P. O'Koye, M. Benham and K. M. Thomas, *Langmuir*, 1997, **13**, 4054.

8. A. Coelho, *J. Appl. Crystallogr.*, 2000, **33**, 899.
